# Reducing patient burden of PROMs in healthcare through advanced computerized adaptive testing stopping rules

**DOI:** 10.1007/s11136-025-04079-7

**Published:** 2025-10-16

**Authors:** Michiel A. J. Luijten, Benjamin D. Schalet, Leo D. Roorda, Lotte Haverman, Caroline B. Terwee

**Affiliations:** 1 Department of Child and Adolescent Psychiatry and Psychosocial Care, Emma Children’s Hospital, Amsterdam UMC location University of Amsterdam, Meibergdreef 9, Postbox 22660, 1100 DD Amsterdam, The Netherlands; 2 Department of Epidemiology and Data Science, Amsterdam UMC location Vrije Universiteit, Amsterdam, The Netherlands; 3 Mental Health, Amsterdam Public Health Research Institute, Amsterdam, The Netherlands; 4 Methodology, Amsterdam Public Health Research Institute, Amsterdam, The Netherlands; 5 Reproduction and Development, Amsterdam Public Health Research Institute, Amsterdam, The Netherlands; 6 Amsterdam Rehabilitation Research Center | Reade, Amsterdam, The Netherlands

**Keywords:** Item response theory, Item reduction, Efficiency, Stopping rules, Methodology, Floor effect

## Abstract

**Purpose:**

Application of computerized adaptive testing (CAT) can improve the assessment of patient-reported health outcomes by reducing patient burden. We aimed to reduce patient burden of CATs further by optimizing a standard error reduction stopping rule (SER; minimum change in SE(*θ*) after each CAT step).

**Methods:**

We extracted PROMIS Anxiety and Depressive Symptoms CAT responses (mean age = 13.7, male = 50.3%) from the Dutch-Flemish PROMIS Assessment Center and estimated theta levels (*θ*) and standard errors (SE(*θ*)) for each step. The default stopping rules were a minimum/maximum of 4/12 items administered, respectively, or a minimum precision of SE(*θ*) < 0.32. We imposed increasing SER thresholds (0.01–0.20) and compared the following outcome criteria: mean efficiency of the CAT (M_efficiency_; 1 − SE*(θ)*^2^/n_items_), mean number of items administered (Mn_items_), the mean SE(*θ*) of all respondents (M_SE(θ)_), and mean T-score difference compared to default stopping rules (M_*∆T*_).

**Results:**

Default stopping rules showed a mean efficiency of 0.88 and1.27, Mn_items_ = 9.98 and8.13, and M_SE(θ)_ = 0.36 and0.38 for respectively the Anxiety and Depressive Symptoms item banks. We optimized the SER value with a differential efficiency function, resulting in shorter, more efficient CATs (Anxiety: mean efficiency = 1.08, Mn_items_ = 5.58, M_SE(θ)_ = 4.24, M_*∆T*_ = 0.04; Depressive Symptoms: mean efficiency = 1.45, Mn_items_ = 4.79, M_SE(θ)_ = 4.15, M_*∆T*_ = 0.58). For participants reporting no problems, this results in fewer items administered, but a decrease in measurement accuracy and biased T-scores, which may be relevant depending on the goal of assessment.

**Conclusions:**

We conclude that the current approach allows us to determine an optimal SER threshold that improves measurement efficiency, especially when floor/ceiling effects are present in the target population. The threshold values will vary depending on the *θ* distribution of the target population and the IRT model parameters.

**Supplementary Information:**

The online version contains supplementary material available at 10.1007/s11136-025-04079-7.

## Introduction

Patient-reported outcome measures (PROMs) that assess a patient’s mental health and functioning in daily life can be important tools to engage patients in their healthcare [1]. PROMs can contribute, for example, to shared decision-making [2] and place value on healthcare when laboratory tests may have more limited relevance to a patient’s daily life [3]. In addition, the use of PROMs in clinical practice can result in improved patient-physician communication and quality of life [4]. Physical, mental, and social health are key conceptual components of overall quality of life [5], and mental health concepts are commonly included in standard PROM profiles [6], typically in the form of self-report depression and anxiety questions.

To develop PROMs, researchers apply psychometric methods that are derived from either a classical test theory (CTT) framework or an item response theory (IRT) framework. PROMs developed under the classical test theory (CTT) framework have the advantage of being easy to score; answers on items are typically summed to produce a total score for each patient. However, all items (i.e., questions) in a given PROM need to be administered, which can lead to excessive burden for patients through repetitive questions [7]. Under CTT, there is no straightforward way to shorten tests without losing reliability and creating incommensurability of scores across PROM versions.

Over the past two decades the application of the IRT framework to PROM development has received increased attention due to its benefits compared to CTT [12]. One potential benefit of the application of IRT is to keep the patient burden lower by applying computerized adaptive testing (CAT) [8]. Specifically, there has been an increased interest in applying CAT to reduce patient burden when assessing mental health in healthcare [9, 10]. Both in research and in clinical practice CATs can provide reliable estimates while administering only a handful of items in several domains of health-related quality of life, such as physical functioning, but also psychological constructs such as anxiety and depression [11]. The Patient-Reported Outcomes Measurement Information System (PROMIS) is a promising measurement system initiated by a cooperative group of scientists from several U.S.-based academic institutions and the National Institute of Health, which consists of a large number of IRT-based item banks to be administered as short-form or CAT [12].

The essence of a CAT based on IRT is that all items (and response options) within an item bank are ordered by item properties known as “item parameters” on a latent trait of interest [13], such as depression. Under the graded response model (GRM, a commonly used IRT model), each item has difficulty parameters or thresholds (β), which represent the level of the construct required to have a higher probability of selecting the adjacent, higher, response category and each item has a discrimination parameter (α), which indicates how well the item discriminates persons among the latent trait. In a GRM, the latent trait is customarily named theta (*θ*), which has a mean of 0 and a standard deviation of 1 in the sample in which the model is estimated. The item parameters can be used to select items from an item bank based on previous responses by applying a variety of available CAT item selection algorithms [14]. For example, if we administer a depression CAT, the first item may be initially administered to optimally discriminate persons from each other near the mean of depression such as “I felt sad”. The response to this item is registered and a first estimation is made of the level of depression of the patient. The next item is selected based on how well it discriminates people near the previously estimated level of depression. Participants that report that they often felt sad may get an item on feeling hopeless (higher thresholds), while participants who report never feeling sad may have received an item on if they wanted to be by themselves (lower thresholds). This process continues until at least one CAT stopping rule is reached.

A common stopping rule for the CAT administration is to stop when the score estimate achieves a threshold of sufficient reliability. Reliability within an IRT framework can be expressed as standard error of theta (SE(*θ*)) and is directly related to the total test information a questionnaire offers for a specific patient at a specific theta:

1 $$SE\left({\widehat{\theta }}_{n}\right)=\frac{1}{\sqrt{I\left({\widehat{\theta }}_{n}\right)}},$$

where the item information (I) is determined by the discrimination parameters ($\alpha$) of the items administered:

2 $$I\left(\theta \right)= {\alpha }_{i}^{2}{p}_{i}\left(\theta \right){q}_{i}\left(\theta \right),$$

where p_*i*_ represents the probability of selecting response category *I* at *θ* and q_*i*_ the probability of selecting any other response category at *θ*. A commonly recommended stopping rule for assessing individuals is 0.32 SE(*θ*), which corresponds to a reliability of 0.90:

3 $$ {\text{SE}}\left( \theta \right) = \sigma \sqrt {1 - r_{xx} } . $$

This stopping rule can result in CATs of varying lengths as respondents (e.g. patients) with different theta levels (e.g., different levels of anxiety or depression) will respond to (partly) different subsets of items, which is why this stopping rule is also known as a variable-length stopping rule. Alternatively, a CAT can stop once a maximum number of items are administered, which is known as a fixed-length stopping rule [15]. Previous research has indicated that a combination of variable-length and fixed-length stopping rules provide the best balance of measurement precision and efficiency [16]. Such a combination of stopping rules is currently implemented in pediatric PROMIS CATs, which typically stop when the SE(*θ*) is less than 0.40 (corresponding to a reliability of 0.80—which is adequate for individual decision-making) or when a maximum of 12 items have been administered [17].

CATs with a combination of a variable-length and fixed-length stopping rule can still administer a relatively large number of items to some respondents. Most PROMIS item banks measure health constructs that are relevant for patients and were developed to perform the best in the range from the mean score of the general population to about two standard deviations in the clinically relevant direction (worse health). Consequently, there are not many items available to provide information for respondents with few or no health problems. Therefore healthy respondents often receive the maximum number of items administered (as the SE threshold is not reached) [18, 19]. This happens often in clinical settings, when CATs are used to screen for problems that are not always present in each individual (e.g., screening for depression or anxiety in pediatric hematology). This results in individuals with no complaints never reaching a reliable estimate to trigger the CAT stopping rule. In educational measurement an often deployed tactic to reduce administrative burden of CATs is by selecting items around a *θ* value used to classify participants as fail/pass [20]. However, this is often not applicable in clinical settings as we are interested in the entire continuum of functioning as the outcomes are also used for monitoring purposes. Nonetheless, we may accept a lower reliability of a healthy measurement and we would like to reduce administrative burden for these patients. In addition, a CAT can run out of items that discriminate well at the level of functioning of a patient, in which case remaining items will contribute only limited improvements in reliability, reducing the efficiency of the CAT.

Given this limitation, it would be relevant to explore if further improvement of these stopping rules can be achieved to avoid unnecessary patient burden. Research into the optimization of stopping rules [15] indicates that either a *minimum change in* SE(*θ*) or an *absolute change in θ* might be useful as an additional stopping rule to reduce administration length. Simulation studies have shown that such stopping rules reduce administration length while retaining reliable measurements in simulation settings [15, 16, 19, 21].

Kallen et al. [19] demonstrated that a change in SE(*θ*) between steps within a CAT can be used as stopping rule during CAT administration [19]. This is also known as the standard error reduction (SER) stopping rule. The SER can be simply defined as:

4 $${SER}_{t}= {SE(\widehat{\theta })}_{t}-{SE\left(\widehat{\theta }\right)}_{t-1},$$

where *t* is the current step in the CAT. If the SER is low, reliability of the measurement does not substantially improve and therefore further administration of items would be inefficient. Kallen et al. [19] demonstrated that a SER stopping rule of 0.01 successfully reduced administrative burden, especially in participants who reported few problems on the domains measured.

More research on the potential benefit of the SER stopping rule is needed. It is possible that increasing the SER value could potentially further shorten a CAT without meaningful loss of precision. Furthermore, a specific threshold for SER may not be optimal or applicable to all item banks [21] as the SER stopping rule is highly dependent on the discrimination parameters, consistency of response patterns, the distribution of theta and the location of items. For example, item banks with few available items to draw from may require a different SER stopping rule to optimize the CAT.

The aim of this study is to develop an approach to optimize the SER threshold when administering a CAT in a sample or population with a large proportion of patients that report the best possible health, using existing CAT data. We will demonstrate this approach on the pediatric PROMIS Anxiety and Depressive Symptoms item banks. Applying this approach to multiple domains will allow us to examine the impact of IRT parameters and theta distribution on the optimal value of the SER stopping rule. The approach described in this study can subsequently be applied in future studies to determine the optimal SER stopping rules for other PROMs administrated as CAT to further reduce patient burden.

## Methods

### Data

We extracted CAT response data (N_anx_ = 3265, N_dep_ = 3304) collected through the KLIK PROM portal for research purposes (www.hetklikt.nu) from April 2020 until November 2020 through the Assessment Center. The data was completely anonymous as only response data to questions (i.e. 1–5) is present in the Assessment Center. Access to this data is limited to a handful of researchers within the KLIK PROM portal team and data access is monitored by the privacy officer of the Amsterdam UMC. All users are aware of who has access to this data. While data was completely anonymous as only response data was extracted, sociodemographic characteristics of the majority of respondents were available as these respondents were part of a study to assess mental and social health of children in the Netherlands during the COVID-19 pandemic [22, 23]. This data consists of a general population sample and a psychiatric sample, consisting of 50.3% boys and a mean age of 13.6 years (range 8–18 years). The default CAT stopping rules of the Dutch-Flemish PROMIS Assessment Center consisted of a SE($\widehat{\theta }$) ≤ 0.32 with a minimum of 4 items administered and a maximum of 12 items administered. Data on item selection and item responses were extracted, so all administration details of the CAT were available for all participants.. Secondary analysis of this anonymous dataset was not considered human subjects research and was exempt from review by the Medical Ethics Testing Committee (METC) of the Amsterdam UMC. The anonymity of the dataset was confirmed by the privacy officer of the Amsterdam UMC. Data included in the dataset was originally collected for research purposes and the participants (and their parents) and researchers gave permission for re-use of the response data. For details on the complete data collection procedure see Luijten et al. and Zijlmans et al. [22, 23]. Both studies were approved by the METC of the Amsterdam UMC.

### Instruments

The PROMIS pediatric Anxiety and Depressive Symptoms V2.0 item banks consist of 15 and 14 items respectively, scored on a five-point Likert scale (ranging from “Never” to “Almost Always”) with a 7-day recall period. The item banks can be administered as a CAT or short-form, each resulting in a comparable theta score by applying standard U.S. item parameters to responses, as per PROMIS convention. The thetas are subsequently transformed to *T*-scores (θ*10 + 50), where 50 is the mean of the U.S. calibration sample with a SD of 10 [17].

### Data manipulation

Post-hoc CAT simulations were performed, where the SER stopping rule was tested for the starting value of 0.01 and subsequently increased with increments of 0.001 until a SER of 0.20 was reached. Each person thus had multiple CAT post-hoc simulations with varying SER thresholds. At each step of each CAT the *θ* and accompanying SE(*θ*) was estimated with the Expected A Posteriori (EAP) estimator [24] using the U.S. parameters, which were obtained from HealthMeasures.

### Evaluation of SER stopping rules

Four outcomes were used (mean efficiency, mean number of items, reliability (mean SE) and mean T-score difference) to evaluate the effect of the SER stopping rule. For each value of the SER stopping rule each outcome was calculated for each respondent using the final *θ* and accompanying SE(*θ*), estimated by EAP. Afterwards the outcomes were averaged across all respondents, resulting in a single (mean) value for each of the four outcomes. The results were subsequently compared to the same outcomes from the default stopping rules. We plotted the results, where the x-axis represented the value of the SER stopping rule and y-axis represented one of the following four outcomes.

First, the efficiency (total test information divided by amount of items administered) was calculated following [18],

5 $$Efficiency=\frac{{(1/SE\left({\widehat{\theta }}_{n}\right)}^{2})}{{n}_{items}}.$$

The higher the mean efficiency, the more information each item adds on average and the more each item contributes to the reliability of the measurement for each respondent. Second, the mean number of items (Mn_items_) administered was counted, where a low mean number of items administered is preferable. Third, the mean SE of final *θ* (M_SE(θ)_) was assessed for each SER stopping rule. The reliability should remain acceptable, therefore a low M_SE(θ)_ is desirable. Fourth, the mean difference between the T-score resulting from the post-hoc CAT simulation with a SER stopping rule compared to the T-score of the default, original CAT stopping rules (without SER) was calculated. A higher mean T-score difference (M_*∆T*_) means that the estimate resulting from the SER stopping rule differs from the T-score resulting from the default stopping rules. The mean T-score difference should be kept to a minimum.

### Subgroup analysis

We purposively split the data into participants with no reported anxiety or depressive symptoms (floor participants) and the rest of the sample and plotted these as separate lines within the aforementioned plots. We expected that the participants with no reported symptoms would benefit most from the SER stopping rule and hypothesized that the stopping rule would not negatively impact the reliability of the other participants.

### Optimizing the SER stopping rule

To optimize the SER stopping rule we calculated the difference in efficiency of CATs by subtracting the efficiency of two subsequent SER stopping rule thresholds from each other (i.e. the differential function of efficiency). If the differential function results in a low value, it indicates that a higher value of the SER stopping rule does not result in relevant improvements in efficiency. We looked specifically at the highest peak of the differential function, which indicates that this SER stopping rule provides the highest increase in efficiency of all possible SER values.

### Effects across range of theta

To investigate the effects of the optimized SER on the reliability and estimates of participants across the entire range of theta, we plotted a smoothed line of *∆T* of the optimized SER stopping rule compared to the default stopping rules across the final estimated theta of the default stopping rules. Similarly, we plotted a smoothed line of the differences in SE (*∆SE)*. This allows us to quickly assess in what regions of theta the optimized SER stopping rule has the largest impact.

### Differences between item banks

These procedures were applied to both pediatric item banks, which differ mainly in their discrimination parameters. Anxiety has overall lower (mean α = 1.57; range α = 1.09–1.89) discrimination parameters than the Depressive Symptoms item bank (mean α = 1.81; range α = 0.74–2.53).

## Results

### Evaluation of SER stopping rules

**Fig. 1:**
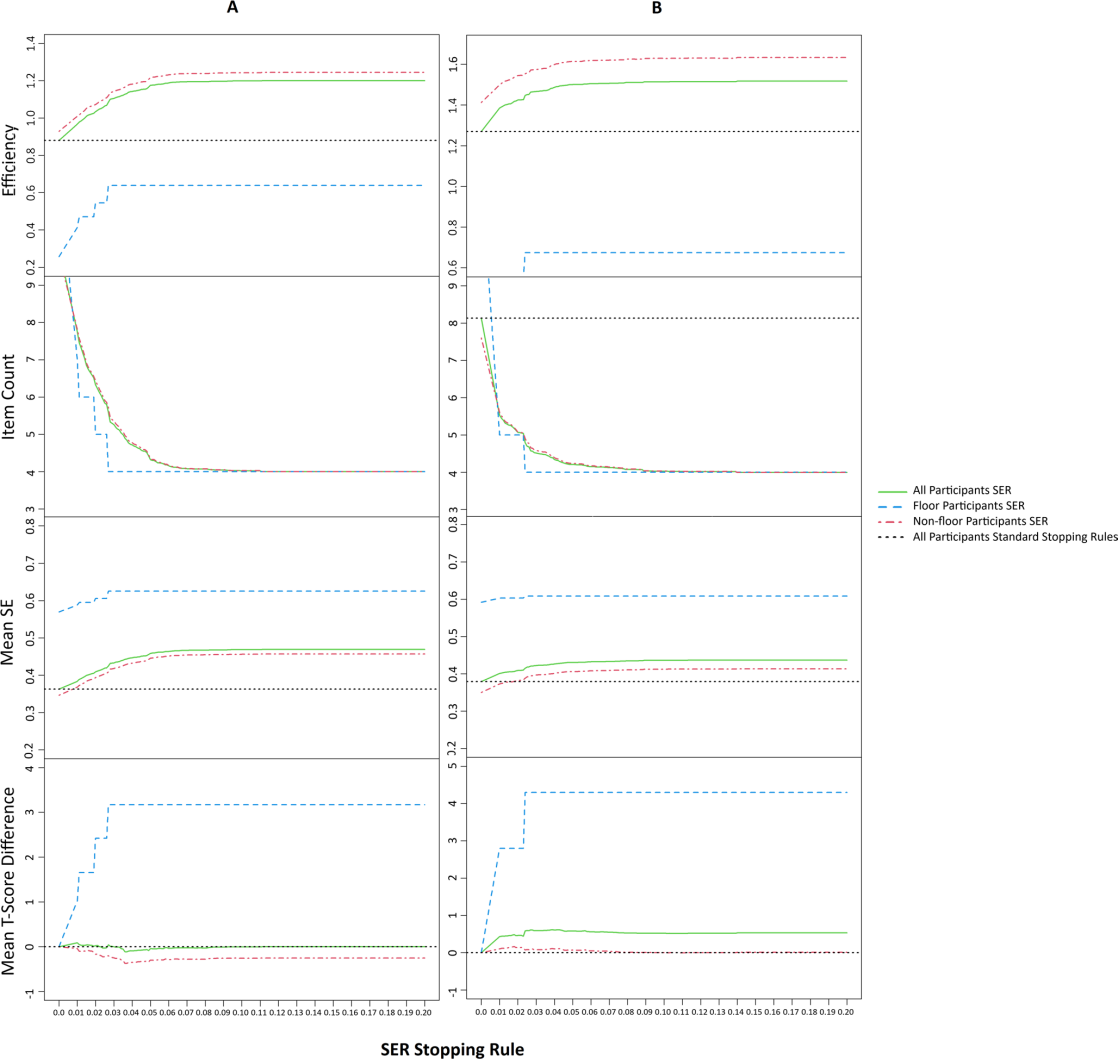
The four criteria plotted for each value of the SER stopping rule for the Anxiety (**A**) and Depressive Symptoms (**B**) item bank

The default stopping rules resulted in a mean efficiency of 0.88 and 1.27, Mn_items_ = 9.98 and 8.13, M_SE(θ)_ = 0.36and 0.38 for respectively the Anxiety and Depressive Symptoms item banks. For the Anxiety item bank 7.2% of the population scored the lowest possible *T*-score of 31.9, for the Depressive Symptoms item bank this was 12.1% with a lowest possible *T*-score of 31.8. For both items bank the efficiency and item counts improved substantially when applying a SER stopping rule. As seen in Fig. 1, the lowest SER of 0.01, resulted in a mean efficiency of 0.97 and 1.38, Mn_items_ = 7.76 and 5.53, M_SE(θ)_ = 0.38 and 0.40, M_*∆T*_ = 0.08 and 0.04, for respectively the Anxiety and Depressive Symptoms item banks. The higher the value of the SER stopping rule, the higher the efficiency of the CAT and the fewer items administered. However, the reliability of measurements decreased and the T-score difference increased with higher SER cut-offs (Fig. 1). Eventually, at a certain height of the SER stopping rule value, each participant is administered the minimum of four items, resulting in a maximum efficiency but in the lowest possible reliability and the largest differences in T-scores (Fig. 1). For details of all SER values assessed and the SDs of the evaluation outcomes, please see Supplementary materials A & B.

### Subgroup analysis

For the participants at the floor (no reported anxiety or depressive symptoms) a clear step-wise pattern can be seen (Fig. 1) for all outcomes. The addition of a SER stopping rule of 0.01 immediately lowers the number of items administered from 12 to 7 and from 12 to 5 for Anxiety and Depressive Symptoms, respectively. However, it also introduces a difference in T-scores compared to the default stopping rules of more than 3 T-score points. Further increasing the SER stopping rule to respectively 0.027 and 0.024 for the Anxiety and Depressive Symptoms item bank resulted in the minimum of four items being administered with a mean T-score difference of 3.2 and 4.3. At these SER thresholds both the Anxiety and Depressive Symptoms item banks were most efficient but least reliable (efficiency = 0.64 and 0.67, M_SE(θ)_ = 0.63 and 0.61, respectively) for participants at the floor.

### Optimizing the SER stopping rule

**Fig. 2:**
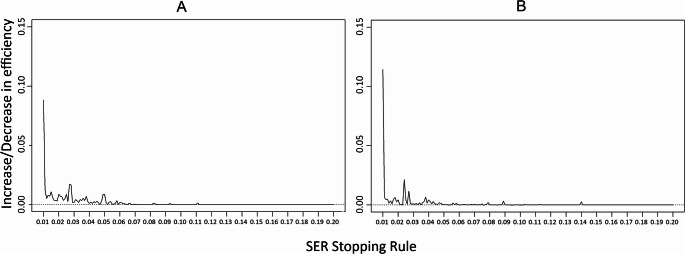
The differential function of efficiency of the Anxiety (**A**) and Depressive Symptoms (**B**) CATs. Each subsequent point in the graph represents the increase/decrease of efficiency compared to the previous SER threshold

The differential functions of efficiency for the Anxiety and Depressive Symptoms item banks are shown in Fig. 2. For both the Anxiety and Depressive Symptoms item bank the highest peak (i.e. improvement in efficiency) was clearly at the introduction of the SER (0.01) stopping rule (mean efficiency = 0.97 and 1.38, Mn_items_ = 7.76 and 5.53, M_SE(θ)_ = 0.38 and 0.40, M_*∆T*_ = 0.08 and 0.43, respectively). For the Anxiety item bank the second highest peak of efficiency improvement was at a SER of 0.027 (mean efficiency = 1.08, Mn_items_ = 5.58, M_SE(θ)_ = 0.42, M_*∆T*_ = 0.04). The second highest peak for the Depressive Symptoms item bank was at 0.024 (mean efficiency = 1.45, Mn_items_ = 4.79, M_SE(θ)_ = 0.42, M_*∆T*_ = 0.58).

### Effects across range of theta

The effects on the reliability and T-scores of the optimized stopping rules (compared to the default stopping rules) were highest for participants at the floor effect (as previously shown) and for participants at the ceiling, for both the Depressive Symptoms and Anxiety item banks. All participants showed some loss of reliability, as fewer items were administered. The relevant plots across the T-score ranges are available in Supplementary Material C.

### Differences between item banks

Overall both item banks displayed similar patterns in terms of all outcome criteria when increasing the SER stopping rule criteria. Applying the initial SER with a criterion of 0.01 to the Anxiety and Depressive Symptoms item bank resulted in a much higher efficiency and fewer items administered compared to default stopping rules. Due to differences in discrimination parameters the effects of increasing the SER stopping rule criteria were more favorable for the Depressive Symptoms item bank than for the Anxiety item bank regarding efficiency and number of items administered. The mean T-score differences were higher at the optimal SER criteria for the Depressive Symptoms item bank (M_*∆T*_ = 0.58) than for the Anxiety item bank (M_*∆T*_ = 0.04). The Anxiety item bank benefited mostly from item reduction at higher SER values for participants with a floor effect, whereas the Depressive Symptoms item bank benefited from higher SER values for non-floor participants as well.

## Discussion

In this study we propose an approach to optimize a variable-length stopping rule to reduce patient burden from CAT assessments when patients do not report any symptoms. Specifically, we showed that the addition of the SER stopping rule reduces patient burden with little to no loss in reliability when assessing (pediatric) anxiety and depressive symptoms. Applying a SER stopping rule successfully reduced the burden of administration for patients, even at the lowest possible SER of 0.01 for both the Anxiety and Depressive Symptoms item banks.

In this study we propose an approach for determining the optimal SER value. By calculating means of efficiency, item count, reliability and difference in T-scores at successive increments greater than SER of 0.01, we identified the most efficient SER stopping rule at 0.027 for the Anxiety and 0.024 for the Depressive Symptoms item banks. At these values, the CATs did not produce any substantial T-score differences or diminishments in reliability for non-floor participants, while reducing the number of items administered for floor participants, when compared to the default CAT stopping rules.

The SER stopping rule is particularly successful in reducing burden for patients who do not report any mental health problems as it can reduce the number of items administered from the maximum of 12 to the minimum of 4, depending on the SER criteria. However, when you introduce or increase the value of the SER stopping rule certain individual patients, especially patients with very low levels of anxiety or depressive symptoms, may no longer be reliably measured and the difference in T-scores compared to the standard stopping rules can be substantial. It is of vital importance that researchers take this into account when using CAT, as a T-score difference of around 3 points is sometimes already considered clinically relevant. For example, when collecting reference data, the mean T-score could vary substantially based on which stopping rules or even which value of SER is chosen, especially when a large floor effect is expected (such as in general population samples).

Previous studies have demonstrated that (PROMIS) CATs provide reliable, valid and above all, efficient measurements of mental health [9, 10, 25]. However, we need to ensure that in settings where a lot of patients may not report (m)any symptoms, these patients are not overburdened with the number of items to complete. We propose an approach to analyze (existing) data to find optimal stopping rules to take this into account. The SER stopping rule can be optimized and applied to accomplish the goal of reducing patient burden.

The optimization of the SER criteria is a separate analytic step to improve CAT performance further and our study serves as a demonstration to be repeated with other banks such as those in PROMIS. It is important to mention that the optimal threshold is dependent on the item bank characteristics, the distribution of thetas in the sample, and the goal of administration. In this study the goal was to optimize the stopping rule to prevent participants with no symptoms from reaching the fixed-length stopping rule. This matched our sample as our theta distribution had a floor effect for the Anxiety and Depressive Symptoms item banks and the goal of administration as it is applied to a general population. For other instruments, there may be other considerations and it would be important to perform simulation studies or use existing data to perform similar optimalisations. For our purpose, previous studies have also shown beneficial effects of a SER of 0.01 [19, 21], which our study demonstrates as well. However, in the case of the two item banks investigated in this study, the application of SER at 0.01 still means that people who do not report any symptoms are being administered five or seven items on average (compared to the minimum of 4). This may not seem like a large difference, but anxiety and depression questions address only two of multiple relevant domains, such as physical function, mobility, fatigue, pain, administered in healthcare settings [6]. In order to assess multiple health domains using CATs, a reduction of almost a third in the number of items administered could be enough to influence healthcare system decisions as to whether or not to include assessment of mental health in routine data collection in clinical practice [10]. For example, if we were to assess all six PROMIS domains included in the PROFILE-25 as CAT, we would reduce the amount of items administered from 72 (6*12) to 24 (6*4) items for patients who have no symptoms or limitations in functioning to report. This would be the same length as the short-forms in the PROFILE-25, while providing more reliable measurements for patients who *do* report health problems. In addition, this study demonstrated that increasing the SER criteria results in barely any negative effects for non-floor participants. Therefore, we consider the optimization of SER values for each item bank to be well worth the additional analytic effort. In fact, in both items banks examined in this paper the optimal values of the SER criteria matched the threshold of SER required for participants that report no symptoms to go from a minimum of 5 to a minimum of 4 items.. It is likely that, when a floor effect is present in the item bank, the SER threshold which reduces the amount of items to the minimum for participants at the floor will be the optimal SER value. The aforementioned change in SE can be deducted in a single simulation given the set of item parameters. When the differential function does not follow this clear-cut pattern of reducing the amount of items administered to participants with floor effects, it will nonetheless only stop the CAT when the items do not contribute any additional (psychometric) information. Selecting an optimal SER threshold in these cases may be more complex as different SER thresholds could result in different regions of theta being measures less reliably. In general, optimizing the SER threshold should not result in a loss of the number of reliably measured participants, but it may reduce reliability overall. This demonstrates that the SER successfully targeted mainly uninformative regions for which we may find it less important to measure accurately, such as in our case when this was limited to the extremes. Similarly, it may be important that the SER retains reliable measurement near a diagnostic thresholds, if this is the goal of CAT administration. Which regions the optimized SER affects can be checked by plotting differences in T-score/SE of the optimized SER value compared to the default stopping rules, across the range of theta with the optimized SER value (see Supplementary Material C).

One remaining issue of the current study is that it is difficult to determine what change in reliability or difference in T-score points is warranted. There are no strict cut-offs, although meaningful change for T-scores has been calculated to be between 2–3 T-score points for most PROMIS item banks, but what is actually meaningful is also dependent on the goal of administration. In our study it is clear that people that report no symptoms have the largest differences in T-scores when changing the stopping rules—but whether this is a meaningful difference is a separate question. In practice, we may find it acceptable that we have reduced accuracy for patients who report no symptoms. However, if a similar T-score difference or loss in reliability is found near cut-off values of severity, we may be less inclined to adapt the new stopping rules.

In this study we did not assess different, additional stopping rules which can be added to the currently defined stopping rules, such as a “best health” approach [19]. In this approach, the CAT stops after a certain number of items have been responded with the highest or lowest response category. While this method successfully deals with participants who appear to be on the ceiling or floor of the item bank when first assessed, it does not take into account people who have a slight variation in responses, but are still in a non-informative or healthy range of the item bank.

There are other, more advanced, methods available that may be applied to obtain similar or even better efficiency of CATs compared to the addition of the SER stopping rule described in this study. Psychological assessment specifically could benefit from multidimensional CATs as domains are often highly correlated, which provides cross-domain information, which results in more efficient CATs [26, 27]. Similarly, the predicted standard error reduction (PSER) would be able to reduce administrative burden even further than SER by predicting when the item bank no longer offers enough information to continue administration of additional items (8, 9). However, there are drawbacks to these advanced methods in practice as it would require an overhaul of existing CAT administration systems and both methods would be more computationally heavy. The advantage of the SER stopping rule is that the required data is already part of the CAT administration procedure and is therefore easier and more practical to implement in existing systems with unidimensional CATs.

When the approach proposed here is applied to other item banks, it will likely provide a variety of optimal SER thresholds, depending upon item bank characteristics. For example, in PROMIS adult item banks, the number of items available (and thus the amount of information available along the range of theta) is much larger and non-informative regions (and floor/ceiling effects) may be less common. Nonetheless, when assessing mental health in medical patients in a hospital setting, there will likely be patients who report no mental problems and would benefit most from the optimized SER stopping rule in comparison to the more conservative SER stopping rule of 0.01. The pediatric PROMIS item banks of Anxiety and Depressive Symptoms have lower discrimination parameters when compared to for example item banks that assess physical health which, based on the results of this study, could indicate that these items banks would benefit even more from the SER stopping rule. Therefore, our advice is to at least use the most conservative SER stopping rule in clinical practice (i.e., 0.01) and, through real data or simulated data, determine the optimal SER criteria per item bank. We conclude that the approach described in this paper can be used to determine an optimal SER threshold in the context of existing clinical data collection programs and that applying this threshold will result in more efficient measurements and less administrative burden for patients.

## Supplementary Information

Below is the link to the electronic supplementary material.

Supplementary Material A. Evaluation of the SER threshold for all tested values of the SER for the Anxiety item bank (for all participants).

Supplementary Material B. Evaluation of the SER threshold for all tested values of the SER for the Depressive Symptoms item bank (for all participants).

Supplementary Material C. Differences in T-scores and SE plotted between standard stopping rules and the optimized SER stopping rule, plotted across the full range of theta.

## Data Availability

Data may be made available upon reasonable request.

## Code availability

Code may be made available upon reasonable request.
