# Supplementary material for: Reducing patient burden of PROMs in healthcare through advanced computerized adaptive testing stopping rules
Source: Qual Life Res. 2025 Oct 16;34(11):3205–14. doi: 10.1007/s11136-025-04079-7 (PMC12681468; doi:10.1007/s11136-025-04079-7)
Supplement: Supplementary file 3 — Supplementary Material C. Differences in T-scores and SE plotted between standard stopping rules and the optimized SER stopping rule, plotted across the full range of theta. [file 11136_2025_4079_MOESM3_ESM.docx]

**
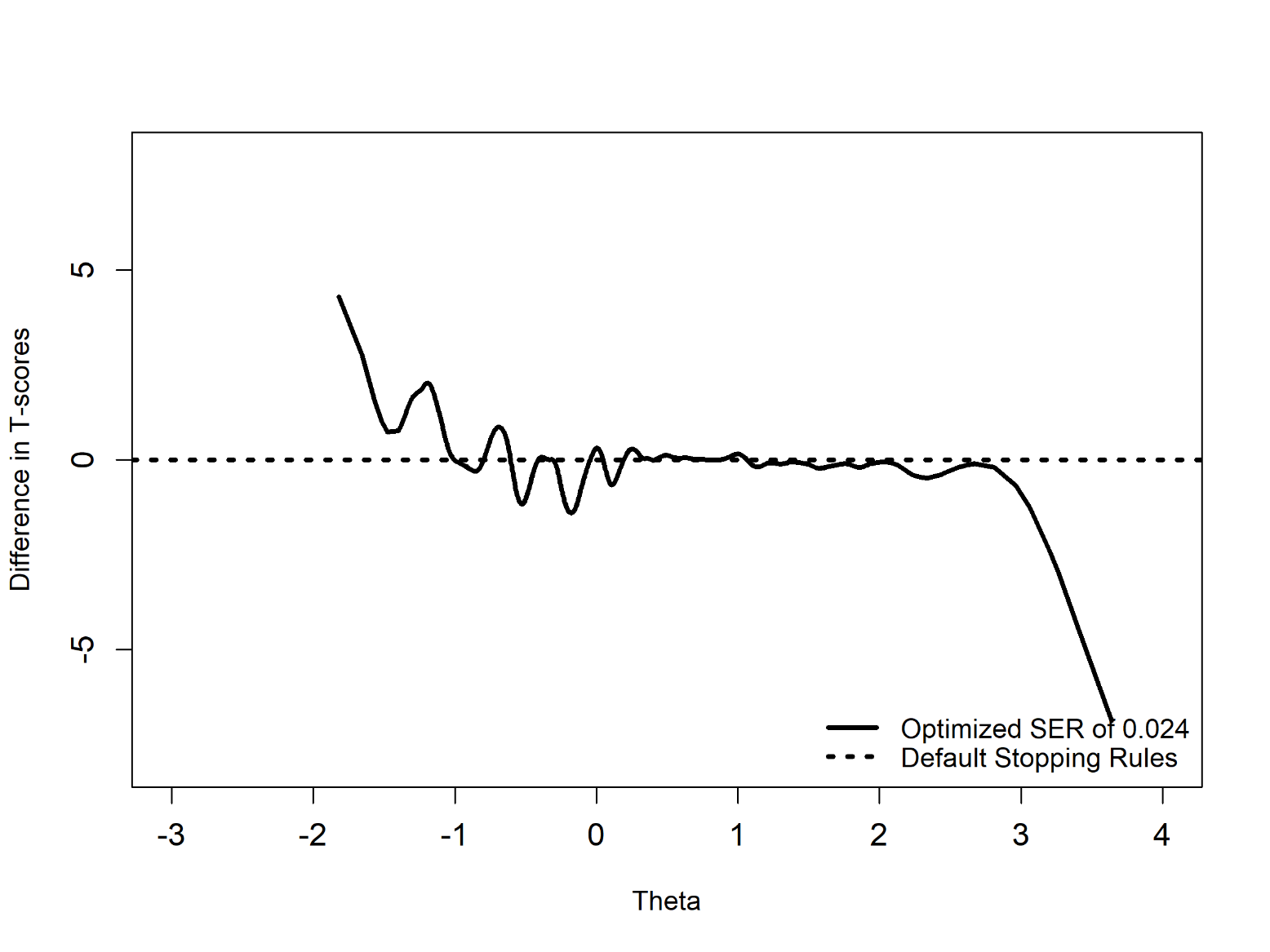
Supplementary Material C**.


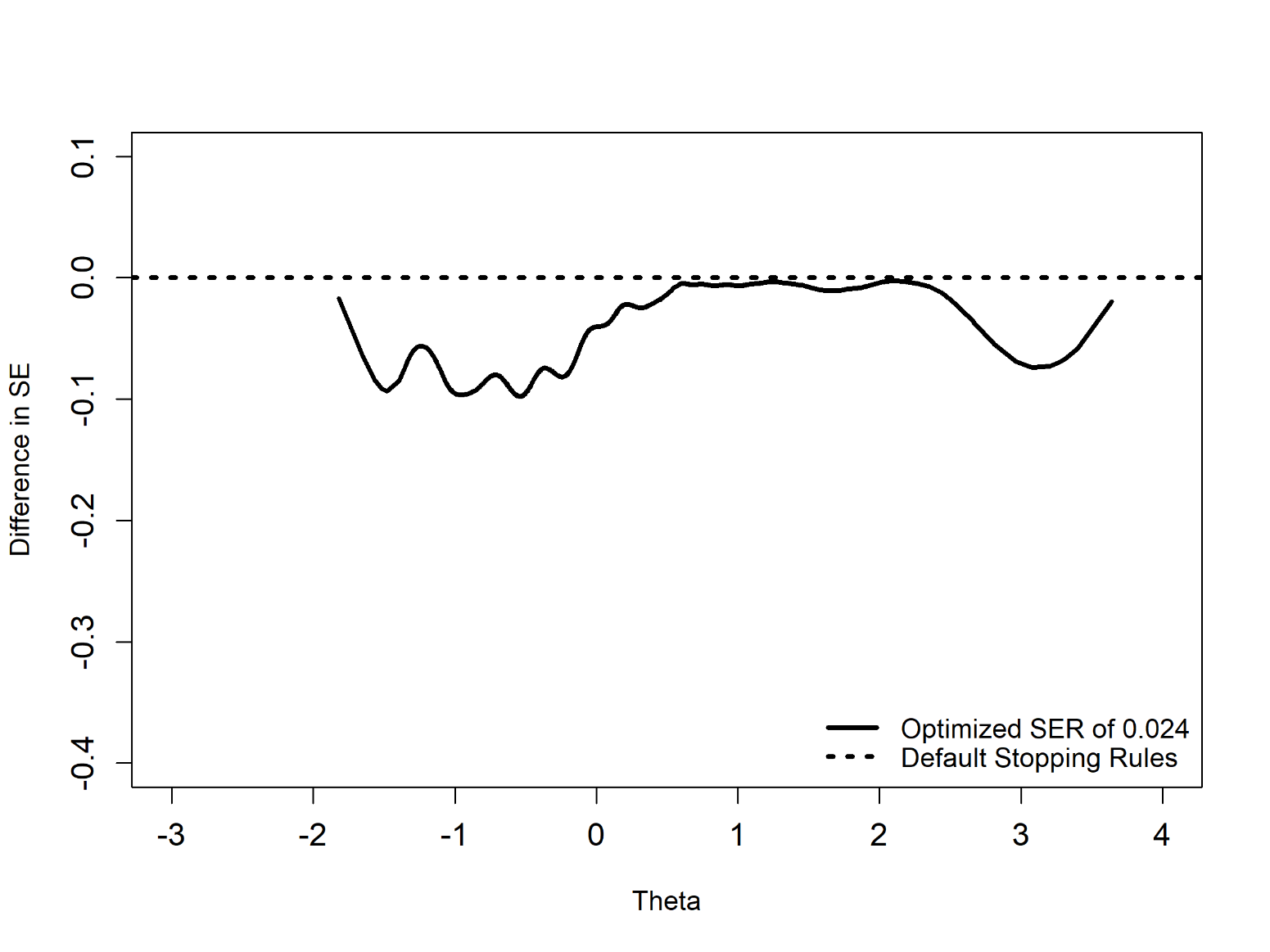


**Figure 1.** Differences in Depressive Symptoms T-scores (upper panel) and SE (lower panel) between the default stopping rule and the optimized stopping
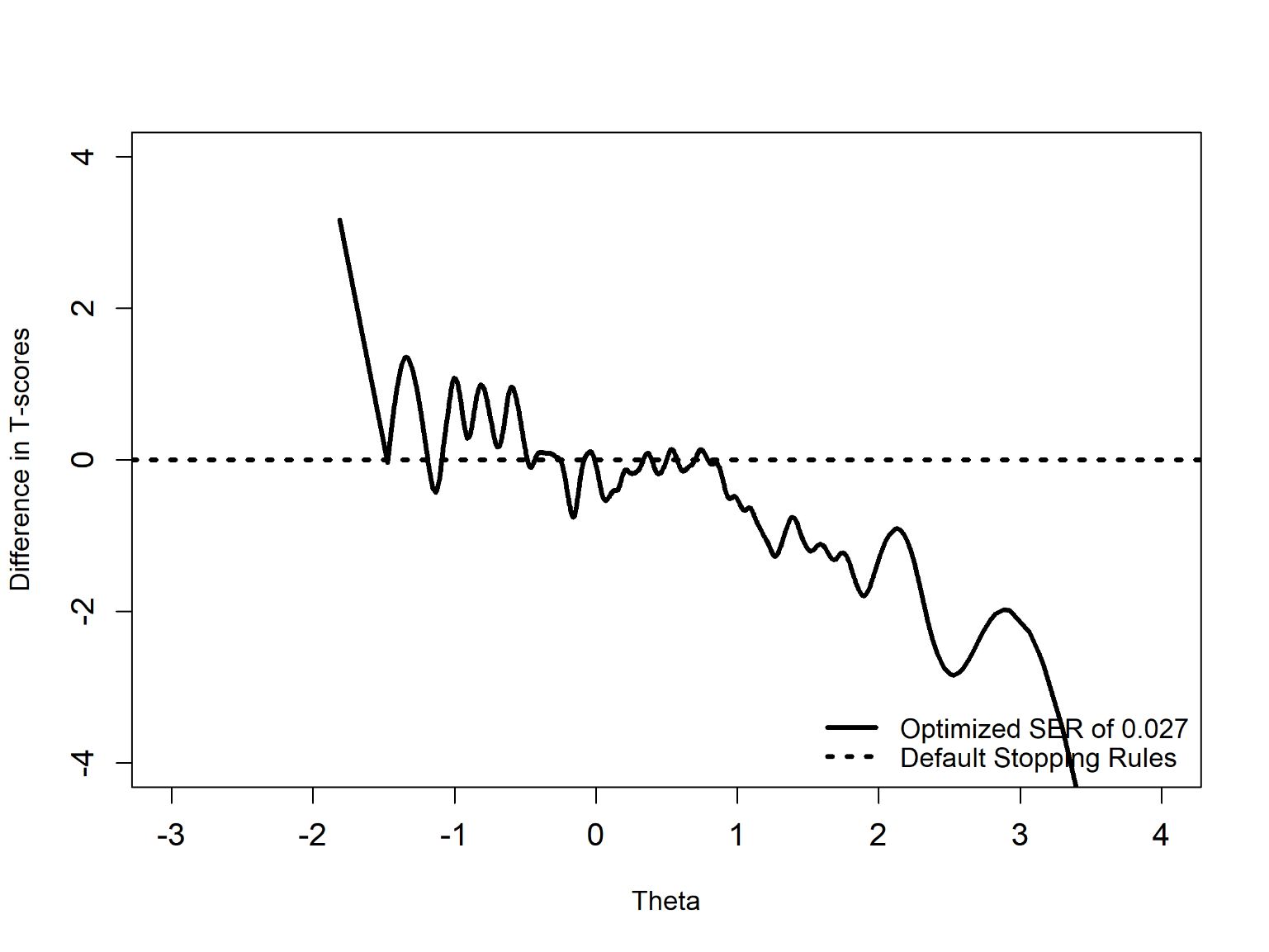
rule smoothed across the range of theta. A higher deviation from the dashed line means the optimized stopping rule results in higher (positive) or lower (negative) T-scores or SE.


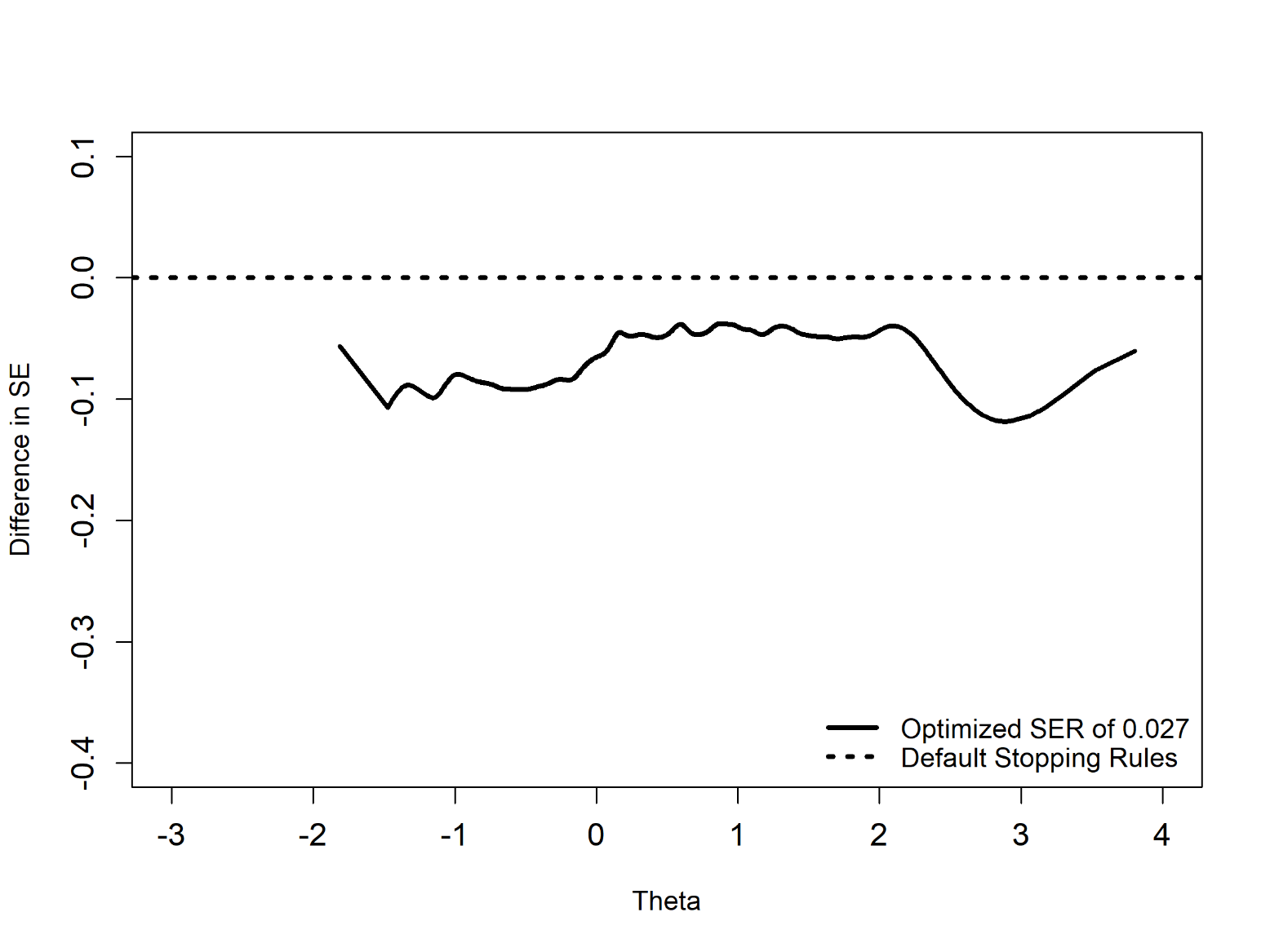


**Figure 2.** Differences in Anxiety T-scores (upper panel) and SE (lower panel) between the default stopping rule and the optimized stopping rule (SER = 0.027) smoothed across the range of theta. A higher deviation from the dashed line means the optimized stopping rule results in higher (positive) or lower (negative) T-scores or SE.
